# Stalk-directed endoscopic submucosal dissection for a giant Brunnerʼs gland gamartoma causing duodenal intussusception with defect closure using three-arm traction clips

**DOI:** 10.1055/a-2739-2639

**Published:** 2025-12-08

**Authors:** Ben-Hua Wu, Jia-Lin Yuan, Li-Sheng Wang, Wen-Biao Chen

**Affiliations:** 1 12387 Department of Gastroenterology, Shenzhen Peopleʼs Hospital, Shenzhen, China; 2 12387 Department of Radiology, Shenzhen Peopleʼs Hospital, Shenzhen, China

A 31-year-old man presented with 6 days of hematemesis and melena. Outside gastroscopy suggested duodenal intussusception with multiple ulcers. Contrast-enhanced computed tomography showed a pedunculated mass arising from the duodenal bulb and prolapsing into the second and third portions of the duodenum, consistent with intussusception ( Fig. 1 ). Endoscopic ultrasound confirmed a bulb-originating lesion with a thick stalk intermittently telescoping into the distal duodenum; mucosal biopsies indicated Brunner’s gland hyperplasia. Endoscopic resection was planned. During therapeutic endoscopy, the lesion was large and highly mobile, precluding secure Endoloop placement because the nylon loop could not be advanced around the mass to the stalk base 1 . A stalk-directed endoscopic submucosal dissection (ESD) strategy was therefore adopted 2 . After the submucosal lift at the stalk, a mucosal incision was made, followed by stepwise dissection. The thick stalk incorporated muscularis propria, necessitating deliberate exposure and division of this layer under direct visualization to achieve complete transection. Final resection was performed with an IT2 knife, and the specimen was retrieved en bloc ( Fig. 2 ). Inspection of the resection bed revealed a focal muscularis propria defect with preserved serosa. Closure was initiated with a three-arm clip (Nanjing Micro-Tech) for precise edge apposition, followed by sequential deployment of multiple through-the-scope clips to ensure complete apposition and reinforcement ( Video 1 ). The procedure was uneventful. Liquids were started on postoperative day 2, and the patient was discharged on day 3. At 1-month follow-up, he remained asymptomatic. Histopathology of the 6.0 cm × 4.5 cm × 2.0 cm specimen confirmed a duodenal hamartomatous polyp consistent with Brunner’s gland hamartoma ( Fig. 3 ). This case highlights that stalk-directed ESD can safely resect giant, intussuscepting Brunner’s gland hamartomas, and that three-arm clip traction plus multi-clip closure can secure a muscularis defect in the thin-walled duodenum, potentially reducing delayed bleeding and perforation.

**Fig. 1:**
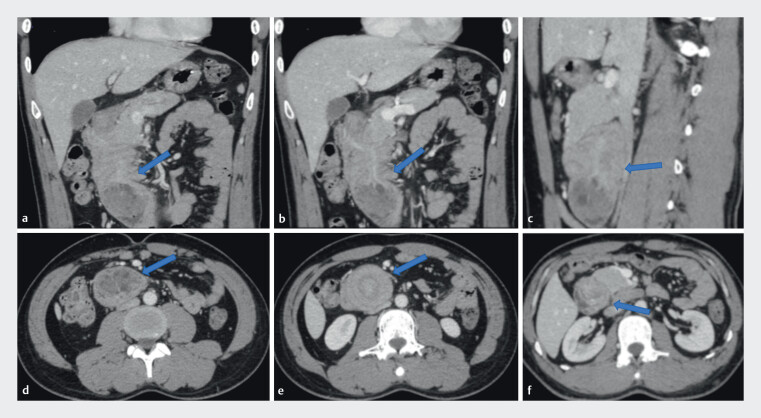
Contrast-enhanced computed tomography demonstrating a duodenal mass. **a** Coronal view (blue arrow); **b** coronal view (blue arrow); **c** sagittal view (blue arrow); **d** axial view (blue arrow); **e** axial view (blue arrow); and **f** axial view (blue arrow).

**Fig. 2:**
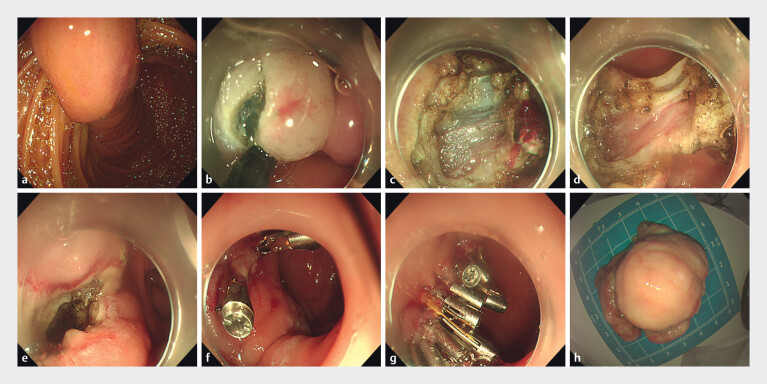
Endoscopic submucosal dissection (ESD) procedure. **a** Endoscopic view of a duodenal lesion with a thick stalk; **b** mucosal incision on the stalk after submucosal injection; **c** partial separation of the stalk exposing muscularis propria; **d** after division of the muscularis propria, residual mucosa and submucosa remain at the stalk; **e** post-resection, the resection bed shows a focal muscularis propria defect with preserved serosa; **f** initial defect reduction with two three-arm clips; **g** additional through-the-scope clips applied to achieve complete apposition and reinforcement; and **h** en bloc resected specimen.

Stalk-directed endoscopic submucosal dissection for a giant Brunner’s gland hamartoma causing duodenal intussusception with defect closure using three-arm traction clips.Video 1

**Fig. 3:**
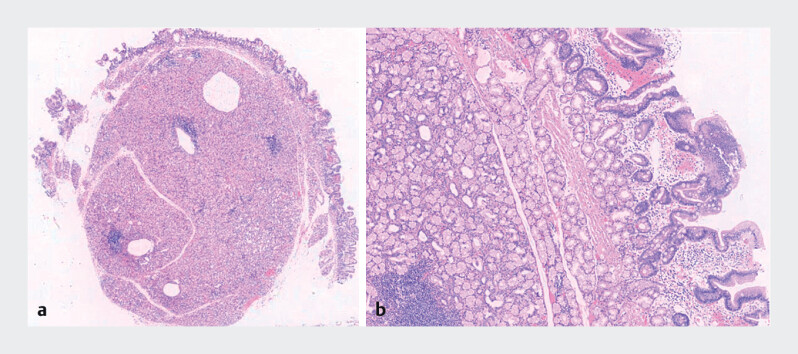
Histopathology of the specimen. **a** Low-power view (10 × 2) and **b** high-power view (10 × 40).

Endoscopy_UCTN_Code_TTT_1AQ_2AD_3AD

## Notes

Endoscopy E-Videos https://eref.thieme.de/e-videos
*E-Videos* is an open access online section of the journal *Endoscopy* , reporting on interesting cases and new techniques in gastroenterological endoscopy. All papers include a high-quality video and are published with a Creative Commons CC-BY license. Endoscopy E-Videos qualify for HINARI discounts and waivers and eligibility is automatically checked during the submission process. We grant 100% waivers to articles whose corresponding authors are based in Group A countries and 50% waivers to those who are based in Group B countries as classified by Research4Life (see: https://www.research4life.org/access/eligibility/ ). This section has its own submission website at https://mc.manuscriptcentral.com/e-videos .
